# “Not pathogenic until proven otherwise”: perspectives of UK clinical genomics professionals toward secondary findings in context of a Genomic Medicine Multidisciplinary Team and the 100,000 Genomes Project

**DOI:** 10.1038/gim.2017.157

**Published:** 2017-10-26

**Authors:** Elizabeth Ormondroyd, Michael P Mackley, Edward Blair, Judith Craft, Julian C Knight, Jenny C Taylor, John Taylor, Hugh Watkins

**Affiliations:** 1 Division of Cardiovascular Medicine, Radcliffe Department of Medicine, University of Oxford, Oxford, UK; 2 National Institute for Health Research Biomedical Research Centre, Oxford, UK; 3 Department of Clinical Genetics, Oxford University Hospitals NHS Foundations Trust, Oxford, UK; 4 Wellcome Trust Centre for Human Genetics, University of Oxford, Oxford, UK; 5 Oxford NHS Regional Molecular Genetics Laboratory, Oxford University Hospitals NHS Foundation Trust, Oxford, UK

**Keywords:** genome sequencing, multidisciplinary team, qualitative research, secondary findings, Medical genetics, Scientific community

## Abstract

**Purpose:**

Approaches to secondary findings in genome sequencing (GS) are unresolved. In the United Kingdom, GS is now routinely available through the 100,000 Genomes Project, which offers participants feedback of limited secondary findings.

**Methods:**

In Oxford, a Genomic Medicine Multidisciplinary Team (GM-MDT) governs local access to GS, and reviews findings. Semistructured interviews were conducted with 19 GM-MDT members to explore perspectives on secondary findings.

**Results:**

While enthusiastic about GS for diagnosing rare disease, members question the rationale for genome screening largely because of lack of evidence for clinical utility and limited justification for use of resources. Members’ views are drawn from diverse experiences; they feel a strong sense of responsibility to act in participants’ best interests. The capacity to return limited secondary findings should be enabled, but members favor a cautious approach that is responsive to accumulating evidence. Informed participant choice is considered critical, yet challenging. Discrimination of variants is considered essential, and requiring of specialist input and consensus. Multiple areas requiring enhanced engagement and education are identified, i.e., for patients, the public, and health-care professionals; at present, mainstreaming of genomics may be premature.

**Conclusion:**

UK experts believe that evidence to inform policy toward secondary findings is lacking, arguing for caution.

**Supplementary information:**

The online version of this article (doi:10.1038/gim.2017.157) contains supplementary material, which is available to authorized users.

## Introduction

Sequencing of the whole genome or exome (GS) is a recently feasible approach to investigating the etiology of diverse rare diseases, offering the potential to identify known and novel gene–disease relationships.^1^ The vast amount of genomic information generated raises numerous questions with ethical, legal, psychological, societal, as well as clinical dimensions.^2^ Approaches to “secondary” findings (SF)—believed unrelated to the presenting condition, including those found “incidentally” (unsought) or “additionally” (through opportunistic screening)—remain the subject of debate, which commentators have suggested hinges on how best to maximize benefits while minimizing harm.^3^

In a research context, Jarvik et al.^4^ have suggested that a “floor” for reporting SF might represent genomic information with important health or reproductive implications, not previously suspected but for which there are proven therapeutic or interventions. They and others^5^ conclude that there is no duty on the part of researchers to search for such information; in a research context, resourcing accredited lab confirmation, variant interpretation, and genetic counseling may be prohibitive.^6^ However, clinical care and research in GS are to date often intertwined, and if there is a “duty to disclose” in research, an argument can be made that there is also an obligation to look.^7^ In this rapidly developing area, researchers and health-care professionals call for professional guidance and regular review;^8, 9, 10, 11^ views are diverse^8, 12^ and dependent on clinical actionability, although many definitional challenges are apparent^9, 13, 14, 15, 16, 17, 18^ (reviewed by Mackley et al.^19^).

Anticipating the advent of “clinical” GS, the American College for Medical Genetics and Genomics (ACMG) published recommendations to screen a list of genes for variants implying risk of potentially life-threatening disease, for which intervention is available, in all individuals undergoing clinical GS.^20^ In contrast, other professional bodies^21, 22^ urged minimization of SF by targeting analysis of genes implicated in the presenting disorder. Counterarguments to the ACMG position focused on ethical issues, such as patient autonomy and the implications of disclosing information about adult-onset conditions to minors, rather than the specific genes included.^23^ Finding some discordance among experts around specific genes or conditions for inclusion, and the relative value of different criteria used for assessing disclosure, Green et al.^24^ acknowledged that it may be “difficult to reach consensus on a specific list of variants that meet a threshold for disclosure.” An updated list is minimally changed,^25^ but policies regarding SF vary by laboratory.^26^ Phenotypic consequences of carrying a disease variant in the “unaffected” population are as yet relatively unknown; recent studies suggest that penetrance and disease burden are less than expected.^27, 28^

In the United Kingdom, genetic investigation of rare disease and cancer is being supplemented by the 100,000 Genomes Project (http://www.genomicsengland.co.uk), recruiting through the National Health Service (NHS) since 2015. Posited as an “NHS transformation project,” the 100,000 Genomes Project aims to capture and store personal genomic and clinical data for combined analysis. Pathogenic variants in genes with proven association with the presenting condition are being reported through established NHS routes. Genomic Clinical Interpretation Partnerships—self-organizing groups of disease specialist researchers and clinicians established as part of the 100,000 Genomes Project—are expected to pursue cases in which no variant in a gene with proven association with the presenting condition is detected. With respect to SF, screening of a small number of genes associated with some cancer predisposition syndromes and familial hypercholesterolemia (subject to change; sought SF are referred to as “additional” findings) is offered to adult participants; screening for recessive and X-linked carrier status is also offered to adults if appropriate. In child participants, optional screening is limited to genes that cause childhood-onset disease.

**Figure 1:**
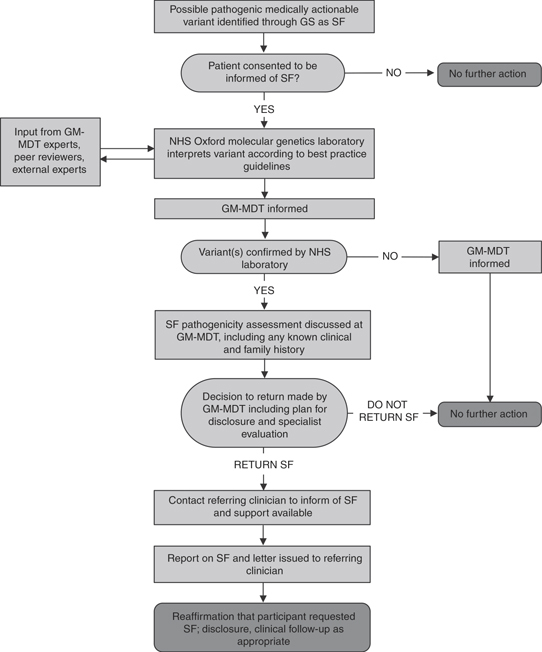
**Pathway for management of SF by the GM-MDT.** GM-MDT, Genomic Medicine Multidisciplinary Team; GS, genome sequencing; NHS, National Health Service; SF, secondary findings.

The current study is based on a rare disease Genomic Medicine Multidisciplinary Team (GM-MDT) established in Oxford in 2014 to maximize potential benefit of translational GS programs,^29^ including, but not limited to, the 100,000 Genomes Project. Other programs within the GM-MDT remit used a purpose-designed research protocol offering options to receive “incidental” findings and, separately, screening of a gene list based on that proposed by ACMG.^20^ The GM-MDT reviews SF according to the pathway shown in Figure 1; it has experience of an established pipeline for GS, as well as of adapting to the nascent 100,000 Genomes Project: two programs with broadly similar aims but distinct policies toward SF. To date, SF in three genes—identified in three participants—have been reviewed by the GM-MDT (Table 1).

**Table 1:** Secondary findings from genome sequencing reviewed by the GM-MDT

**Gene**	**Condition associated**	**Variant interpretation**	**GM-MDT decision**
*BRCA2* (heterozygous)	Breast/ovarian cancer	Highly likely pathogenic	Report
*RYR1* (heterozygous)	Malignant hyperthermia	Uncertain significance	No report
*KCNQ1* (heterozygous)	Long QT syndrome	Likely/highly likely pathogenic	Report

GM-MDT, Genomic Medicine Multidisciplinary Team.

This study aimed to inform the current debate around genomic SF by exploring and collecting rich, contextualized data on the perspectives of GM-MDT members of the generation and disclosure of SF, in the context of explicit policy developed for a large-scale clinical GS program in the United Kingdom.

## Materials and methods

### Data collection

The GM-MDT has met monthly since April 2014; it comprises invited members who are medical doctors across a broad range of specializations (adult and pediatric), genetic counselors, NHS clinical scientists/bioinformaticians, research scientists with expertise in genomics, and study coordinators. Thirty-six GM-MDT members were invited to participate in a semistructured interview by individual e-mail; this represented all members during the recruitment period. Interviews were conducted between August 2015 and April 2016; thus, 16 GM-MDT meetings had occurred by the time of the first interview. Data-driven, qualitative methods were chosen to allow members to reflect, and to expand on their responses. Interviews were conducted face to face by E.O. (a GM-MDT member), and lasted between 40 minutes and 2 hours. An interview guide (Supplementary Materials online) was devised from a review of the literature, and modified slightly as interviews progressed. Written informed consent was obtained under the Molecular Genetic and Analysis and Clinical Studies of Individuals and Families at Risk of Genetic Disease study protocol, approved by the West Midlands Research Ethics Committee, reference 13/WM/0466.

### Data analysis

Interviews were audio recorded, professionally transcribed verbatim, and subjected to thematic analysis.^30^ Interview transcripts were initially coded and analyzed iteratively, both manually and using NVivo 11 (QSR International, Melbourne, Australia) by E.O. and M.P.M. After analysis, the transcripts were reread to verify concordance with the data and final themes agreed by consensus. Final analysis was presented in writing to members and minor modifications were made. In the following section, selected quotes are used to illustrate themes; study participants, assigned codes, are referred to as “members” to distinguish from GS “participants.” Analysis of interview data focusing on the functioning of the GM-MDT has been reported elsewhere.^29^

## Results

Nineteen members took part (Table 2). The full range of professions was represented; broad role descriptors are used to protect identities. At the time of interview, two members—both clinical geneticists—had disclosed SF from gene panel or research GS on more than one occasion. Four major themes were derived from the analysis (Table 3).

**Table 2:** Demographics of interviewees (*n* = 19)

**Category**	***n***	%
Gender		
Male	12	63.2
Female	7	36.8
Role		
Clinical (genetics; including genetic counselor)	5	26.3
Clinical (nongenetics: main specialty other than clinical genetics)	8	42.1
Nonclinical (clinical scientist, researcher, study manager/ coordinator)	6	31.6
Consented patient participants to genome sequencing (and/or exome sequencing)		
Yes	7	36.8
No	12	63.2
Years’ experience (self-defined as relevant to role in GM-MDT)		
5 or less	1	5.3
6–10	3	15.8
11–15	3	15.8
16–20	6	31.6
21–25	2	10.5
26 or more	4	21.1

GM-MDT, Genomic Medicine Multidisciplinary Team.

**Table 3:** Primary and secondary themes derived

**Rationale for search for secondary findings in clinical genome sequencing**
• Support for search and disclosure of limited secondary findings with high predictive value, and carrier status
• Requirement for diverse evidence of clinical utility
• Caution about overinterpretation
• Inadequate justification for use of resources
**Discriminating between secondary findings**
• Disclosure decisions should be at variant level
• Disclosure decisions should be independent of patient specific factors, but contextualized
• Need for data informing how case selection changes penetrance estimates
• Concept of family-based penetrance
**Responsibilities: professional, societal, and personal**
• To enable limited disclosure while enhancing evidence base
• To prepare and protect participants
• To engage and educate widely
• To continue to provide risk assessment and family history–directed panel testing
**Feasibility of informed consent**
• Patient choice and informed consent essential
• Consent should be broad, with capacity to change preferences

Bold type denotes primary theme.

### Rationale for search for SF in clinical GS: why screen, and for which conditions

While enthusiastic about the application of GS to rare disease and considering it potentially transformative for health care, some members suggested that “secondary findings make it messy” (M28, M17). Several felt strongly that the current evidence base is too limited to consider nonprimary findings as other than a research question:

*The fundamental issue is we don’t have enough data to be able to say reliably what the implication of these variants is on the whole*. (M48, nonclinical)

Some members articulated a conceptual distinction between research and clinical practice, for example, “if A causes B that’s fine, if A could cause D that’s still research” (M28), and pointed out that genome screening, at present, is unorthodox:

*It’s like trying to take stuff away without any body of knowledge, suddenly just delivering this into the clinic. You wouldn’t get away with it anywhere else.* (M49, clinical, genetics)

Several were wary of offering genome screening “just because we can,” and some favored restricting analysis to virtual gene panels, intentionally minimizing nonprimary findings. Most members favored a cautious approach, stressing the need to collect data, reflect, and evolve with experience:

*We’re increasing our potential to overinterpret maybe, even with the primary findings but also we could be overzealous in the secondary findings and it doesn’t sit so easily really…keeping it small, limited, to explore and find out what all the limitations are would be a way forward*. (M25, nonclinical)

The required evidence base was variously considered to include understanding the disease-predictive value of “pathogenic” variants when identified as SF, clinical decision-making pathways, and understanding what use people might make of SF after disclosure.

Most members who were aware of debates around approaches to SF agreed that there is a need for a consensus list of genes—international, or at least national—with some recognizing the need for broad societal input. Most considered that any such list should be restricted to medically actionable, penetrant, and serious conditions, acknowledging inevitable inconsistencies resulting from “drawing a line” when definitions are subjective. A small number of members considered a broad definition of “actionability,” to encompass conditions that may not be medically actionable but of which awareness might influence life planning. Some saw a clear rationale for opportunistic screening if/when the presence of a variant could be viewed as an early sign of disease, when there were clinical tests to inform risk and intervention, allowing treatment of phenotype rather than genotype:

*The cardiac ones are relatively easy…we’ve got quite good tools for monitoring people and evaluating whether there really is a risk or not.* (M32, clinical, nongenetics)

Conversely, while recognizing the rationale for their inclusion, one member perceived that including cancer predisposition genes could cause anxiety among colleagues whose role might include disclosure of SF to their patients:

*Everyone’s scared of the cancer genes but they’re on there because there are screening plans and more people these days die with cancer than of cancer.* (M26, clinical, genetics)

Some discussed the range of possible risk management interventions that might be warranted, suggesting that the burden of proof would need to be high when intrusive, irreversible interventions might be considered, or when no intermediate phenotype is discernible.

Nongenetics professionals were less familiar with recent debates around approaches to SF in the literature and beyond, emphasizing informed consent as the main determinant for the chosen approach.

Information that could inform reproductive decisions was considered actionable by all members, especially *in situations* in which the manifest condition affects a child and the parents likely to be planning further children. Some envisaged dilemmas when partners changed, or made divergent decisions.

Some mentioned ways in which the efficacy of clinical genetics in the NHS is resource-limited, and suggested directing resources to alternative approaches to early detection or prevention of serious diagnosable diseases, such as improving access to family history–directed gene panel testing, or preconception carrier screening for serious untreatable conditions. Some expressed strong feelings about a perceived inadequacy of resources for GS infrastructure, and the impact this has on capacity both to ascertain and recruit families. Inadequate justification for spending resources on screening for SF was cited as reason for restricting investigation to primary findings, and one member questioned the rationale for genome screening only people eligible for GS:

*The reality is that if we weren’t doing this Genomics England project*—*I mean there’s loads of people that are not going to be part of Genomics England, all of whom have got de novos or recessives or something.* (M17, clinical, genetics)

### Discriminating between SF: which to disclose

While members agreed that, if the rationale for some degree of genome screening is accepted, there should be a consensus gene list, they also felt strongly that reporting decisions needed to be at the variant level:

*If you just put the gene name on you get the problem. The key would be only the rock-solid variants in that gene, and that’s tricky because in some genes that’s just variants which have got a good established literature, experimental data, segregation.* (M32, clinical, nongenetics)

Members drew on experience of genes relevant to diseases of which they had specialist knowledge, stressing the difficulty of interpreting primary findings, and that interpretation usually addresses the question, “does this variant account for this patient’s phenotype?” Some noted that the scientific literature contains inaccuracies and errors, based on unacceptably low burdens of proof, highlighting the need for expert interpretation. Some recounted experiences of nonexperts ascribing causation to variants in genes with “known” association to the patient’s disease, that were present at high frequencies in population databases, or failed to segregate. Many expressed misgivings or anxiety about the harms that could be caused by erroneous interpretation.

In addition to concerns about variant interpretation, members stressed the importance of collecting data relating genotype with phenotype in the unaffected population, to inform penetrance estimates. Some suggested that penetrance in high-risk families may be modulated by other unknown factors, making it impossible to provide accurate risk counseling and invoking the concept of “family-based penetrance.” There was consensus that limited, highly likely pathogenic variants should be reported irrespective of patient-specific factors; recommended follow-up could however be context-dependent, for example taking into account the patient’s age, sex, family history, and where possible, clinical findings.

If it is possible to distinguish the more cautious members from the less, this appeared to center on perceptions of patients’ “tolerance of uncertainty.” For some members, risk estimates remain too theoretical to be applied beyond the research domain, while those members more favorable toward genome screening believe that a limited list of variants should be considered at present—one that is curated to capture accumulating data.

### Responsibilities: professional, societal, and personal

Members frequently took time to consider responses, drawing on both professional and personal experiences and interactions; they envisaged significant benefits but also significant harms. Members expressed nuanced views: a clearly perceived sense of a duty to warn patients—related to clearly pathogenic variants associated with beneficial medical management—could coexist with strong views about the need for caution. Some expressed the view that the predominant responsibility among the genomics community is to develop an evidence base and learn from experience.

An increasing appreciation of genomic complexity was mentioned by members across all professional roles, and some described how they had become increasingly aware of ethical arguments for and against generation of SF. Interviewees raised concerns—sometimes expressed as anxieties—including: the possibility that many findings of potential but unclear significance might be unearthed, that clinical laboratory confirmation might occur without a clear pathway for disclosure, and that highly predictive information could not be disclosed due to lack of consent. This evoked strong views that disclosure of SF should be enabled within GS protocols.

Subjective reflections contributed to views, particularly for members who did not have clinical roles. Some acknowledged that decision making would be difficult, stressing the familial implications, envisaging a sense of responsibility toward their own relatives and considering how best that might be fulfilled:

*It’s a tough one… to begin with I would’ve just said no, I don’t want to know but I have [relatives] so that could obviously affect [them]…I suppose those sorts of arguments have been in the public domain over the last 2 or 3 years really have caused me to think more about it.* (M18, nonclinical)

Members considered they have a responsibility to prepare and protect patients and families from harm, i.e., not to “burden families with unexpected and unwanted information” (M13) that has the potential to provoke potentially long-term worry and clinical interventions “in the anticipation of risk” (M21), and perhaps insurance and employment discrimination. Hypothesizing about personal GS, (some participants had, or knew of people who had had GS in a nonclinical setting) members who considered themselves able to interpret genomic data wished to access the whole data set, whereas members with less bioinformatic competence generally thought they would want actionable results “in response to a clinical question or proposed treatment” (M21); “keep it simple” (M28).

Notwithstanding the limitations of a family history–based approach to diagnostic genetic testing, particularly for cancer predisposition, some were wary of SF being used to circumvent traditional risk estimation, and considered it the responsibility of referring clinicians to take a full family history and refer appropriately. Some noted that discussions about SF could prompt recall of family diagnoses.

Members agreed that disclosure should be initiated by a health-care professional known to the participant who should ensure that referral to an appropriate specialist occurs within a short time frame. Most viewed responsibility to relatives as being shared with individual participants; they saw no major differences from traditional genetic risk communication, although one reasoned that communication might be more challenging in the absence of a family history. Members were appreciative that the GM-MDT enabled shared responsibility for all aspects of disclosure, but noted that enhanced engagement with, and education of, the wider clinical community were required. Responsibility might extend to promoting realistic concepts of the limitations of genetic testing:

*Where you can say the medical community is at fault is that, within the media, and pushed by some commercial companies, one is constantly bombarded with a sense of genetics being deterministic, predictive, and having all these exciting consequences for future health and health management, which I don’t believe myself…the public are often extremely receptive to these notions and think the quality of information is higher than is actually the case.* (M20, clinical, genetics)

Perceived underappreciation of the complexities of genomic information, considered widespread among health-care professionals, led some to suggest that mainstreaming of GS is premature.

### Feasibility of informed consent

Members unanimously agreed on the importance of patient choice, and considered that informed consent was essential to maintain patient trust; they suggested that the possible range of outcomes, likelihood, and consequences for the participant and their family should be covered. Perceived challenges to informed consent were multiple: the requirement for a complex discussion of risk, the length and complexity of the documents, lack of professional understanding, time required, and other pressures on resources. It was recognized that the stated potential for the gene list to change was realistic and therefore consent needed to be broad, underpinned by a conceptual appreciation of possible findings, and that any implied risk would be actionable. It could be difficult to assess how much to focus on SF when the likelihood was small for any given patient.

Members considered that the quality of informed consent was probably variable, and reliant on the “professional insight of the one who’s doing the consent” (M22). Members who envisaged that they would be in the position of disclosing SF emphasized the attention they devote:

*I almost felt myself feeling the need to make sure he understood the negative implications […] I do feel that they’re also not going to appreciate it if I phone them up afterwards and say we found this gene that you didn’t even expect.* (M15, clinical, nongenetics)

Members reflected on varied consent discussions with participants; they had developed strategies that included gauging participant level of understanding and giving examples to promote a broad awareness rather than discussing individual conditions in detail. Although some thought that effective informed consent was possible, if time consuming, others were skeptical, pointing out the vulnerability of undiagnosed patients and families, focus of attention on finding a cause for the manifest condition, and limitations of public understanding. Some felt that participants overestimate the yield of SF and do not appreciate their limited value. Many members had not personally taken consent; some clinical members had delegated this to others, especially when they worked alongside or could refer to genetic counselors.

Some frequent consenters noted that people not infrequently change their minds about receiving potentially predictive genetic information, and several members considered the life stage–specific relevance of genomic information—for example, that carrier status might be appropriate and late-onset conditions less so as a young adult, reversing in middle age when reproductive decisions had already been made. Some suggested that decisions should be made in conjunction with relatives. Some noted that since attitudes continue to evolve, both personally and within the genomics community, it was reasonable to expect that participant attitudes might be subject to similar or other influences with developing knowledge and understanding: dynamic forms of consent were considered an aspiration, albeit resource intensive.

## Discussion

We present findings of a study exploring UK genomics experts’ perceptions of secondary genomic findings in a clinical setting, in the context of a multidisciplinary team and the 100,000 Genomes Project. Analysis shows that genomics professionals are enthusiastic about GS and its potential, but are uncertain about the utility of SF given the available evidence base, and the rationale for genome screening. Members of the GM-MDT perceive a strong sense of responsibility to protect patients and families, but are not clear how best to enact this at present. In the current evidence vacuum, members’ views are informed by a range of sources: discussions with patients and with colleagues within and outside formal structures, comparable contexts, and personal and social interactions. A limited screening approach is considered optimal, promoting patient choice while restricting disclosure to clear pathogenic variants while concentrating resources on building an evidence base. Findings align closely with the views of Burke et al.^31^ and Janssens^32^ who argue for continued debate and development of an evidence base including potential harms.

The concept of personalized medicine is gaining ground, yet gaps in interpretation and translation remain. Professional expertise and judgment will be required to ensure that decisions about variant interpretation, disclosure, and consequent management strategies are appropriate and responsible.^33^ Key questions concern interpretation of variants, and penetrance estimates. In this study, members emphasize the importance of accurate, informed variant interpretation; even with specialist knowledge, laboratories reach discordant conclusions^34^ and consideration should be given to establishing an SF variant list in addition to a gene list. The penetrance of pathogenic variants in the wider population—among individuals who have not been selected for family history of that disease—remains unknown. Furthermore, “penetrance” is a population-level descriptor, which may be of limited value for predicting risk in an individual receiving an SF. ACMG emphasized the need for “contextualizing” risk estimates at disclosure with age and family history,^20^ and we concur: in an individual family, unknown variables may modify disease expression.

Evidence-based guidelines on use of genetic tests in clinical practice require systematic assessment of clinical utility; this can be considered a concept of net benefit,^35^ taking into account clinical endpoints but also psychosocial, ethical, legal, and social factors. In assessing clinical utility, as well as understanding the predictive value of variants identified as SF, it will be important to understand sequelae of disclosure^36^ and of “living at risk.”^37^ In the present study, experts vary in their perceptions of patient tolerance for uncertainty; most who discussed this had clinical roles. Greater perceived tolerance for uncertainty tended to associate with a more favorable attitude toward SF; if substantiated through further research, this would be consistent with findings that intolerance for uncertainty is a predictor of distress on receipt of genetic information of uncertain value.^38^

The ACMG list does not include autosomal-recessive or X-linked genes. In the present study, members had less difficulty in agreeing that such findings be sought with a view to return. Although this approach could lead to ethical tensions arising from overlap with dominant conditions that are as yet not medically actionable but could be used to inform reproductive decisions, optional screening for carrier status should be considered in GS protocols.

This study highlights several drivers for engagement and education, both public and professional. Members unanimously consider that informed consent and participant choice are critical, yet have reservations about patient understanding and appreciation of the limitations of genetic information. This is relevant at recruitment, and in the event of disclosure. Multiple issues should be covered during consent for clinical GS,^39^ but participant “choice” with regard to SF may be less than straightforward.^40^ Effectively conveying the information required to promote informed consent requires professional insight—while genomics has been steadily gaining ground in mainstream medicine (likely accelerated by the 100,000 Genomes Project), health-care professionals delivering genomics need to be equipped with basic knowledge, as well as routes to specialist expertise.

### Strengths and limitations

Study subjects are diverse in specialty, role, and experience and represent views on a publicly funded, large-scale clinical GS program distinct from those reported on to date.^19^ However all participants are employed by the University of Oxford or Oxford University Hospitals Trust; genomics professionals at other centers in the United Kingdom, and internationally, may have different perspectives.

### Conclusions

Findings show that health-care professionals and researchers engaged in genomic medicine in Oxford believe that present policy around SF should be considered conditional; evidence is required to understand variant pathogenicity and penetrance in diverse populations, as well as impacts of disclosure on individuals, families, health-care professionals, and on health-care systems. While evidence accumulates, we advocate for a cautious, limited approach, and urge wide engagement and education with the public and health-care professionals.

## Supplementary information

Supplementary Information (DOCX 16 kb)
