# Supplementary material for: “Not pathogenic until proven otherwise”: perspectives of UK clinical genomics professionals toward secondary findings in context of a Genomic Medicine Multidisciplinary Team and the 100,000 Genomes Project
Source: Genet Med. 2017 Oct 26;20(3):320–8. doi: 10.1038/gim.2017.157 (PMC5880578; doi:10.1038/gim.2017.157)
Supplement: Supplementary file 1 — Supplementary Information (DOCX 16 kb) [file 41436_2018_BFgim2017157_MOESM12_ESM.docx]

**GM-MDT Member Interview Guide**

**Demographic information**

1. What is your role: clinical (genetics), clinical (other, non-genetics) non-clinical researcher, clinician/researcher, clinical scientist
2. How many years relevant experience do you have?

**Views and experiences of SF and genomic medicine**

1. What are your opinions on informed consent processes (ie. written material and consultation) for WGS, specifically MGAC and 100K? How far does it facilitate decision making, could it be improved?
2. Have you personally consented patients/family members? If so, how did you find this process? Do you think patients will be adequately informed should a secondary finding (SF) need to be reported (as incidental or additional)? Have you changed anything about your approach to informed content based on experience?
3. Do you perceive a tension between clinical duty of care and research agenda in generation of SF?
4. Do you think informed consent choices, such as about secondary findings, should always be respected? Why/why not?
5. What factors are important in reporting decisions re SF?
6. What do you see as the pros and cons of having a list of genes that are analyzed for variants? Do you think the ACMG list is reasonable, why/not?
7. Where do you feel responsibility to patients you are involved with lies in the weighing of benefits/harms in secondary findings?
8. Do you perceive any responsibility to patients’ relatives re SF? If so, how would you manage this/do you think this should be managed?
9. If you were a participant or parent, how do you think these opinions be any different?
10. How do you think disclosure of secondary findings should be managed? How much should disclosure be contextualized for the patient?
11. How do you think approaches to genomic information generation might change as genomic medicine becomes more routine?
